# Effect of plant produced Anti-hIL-6 receptor antibody blockade on pSTAT3 expression in human peripheral blood mononuclear cells

**DOI:** 10.1038/s41598-023-39106-5

**Published:** 2023-07-24

**Authors:** Namthip Kaewbandit, Ashwini Malla, Wanuttha Boonyayothin, Kaewta Rattanapisit, Thareeya Phetphoung, Nuttapat Pisuttinusart, Richard Strasser, Rattana Saetung, Supannikar Tawinwung, Waranyoo Phoolcharoen

**Affiliations:** 1 Center of Excellence in Plant-Produced Pharmaceuticals, Chulalongkorn University, Bangkok, Thailand; 2 Department of Pharmacognosy and Pharmaceutical Botany, Faculty of Pharmaceutical Sciences, Chulalongkorn University, Bangkok, Thailand; 3 Graduate Program of Pharmaceutical Sciences and Technology, Faculty of Pharmaceutical Sciences, Chulalongkorn University, Bangkok, Thailand; 4 Baiya Phytopharm Co., Ltd, Bangkok, Thailand; 5 Department of Applied Genetics and Cell Biology, University of Natural Resources and Life Sciences, Vienna, Austria; 6 Department of Pharmacology and Physiology, Faculty of Pharmaceutical Sciences, Chulalongkorn University, Bangkok, 10330 Thailand; 7 Cellular Immunotherapy Research Unit, Chulalongkorn University, Bangkok, Thailand

**Keywords:** Molecular biology, Plant sciences

## Abstract

As a response to invasion by pathogens, the secretion of interleukin 6 (IL-6) which is a cytokine, activates IL-6/JAKs/STAT3 intracellular signaling via., phosphorylation. Over expression of pSTAT3 induces IL-6 positive feedback loop causing cytokine release syndrome or cytokine storm. Plants have gained momentum as an alternative expression system. Hence, this study aims to produce mAb targeting human IL-6 receptor (hIL-6R) in *Nicotiana benthamiana* for down regulating its cellular signaling thus, decreasing the expression of pSTAT3. The variable regions of heavy and light chains of anti-hIL-6R mAb were constructed in pBYK2e geminiviral plant expression vector and transiently co-expressed in *N. benthamiana*. The results demonstrate the proper protein assembly of anti-hIL-6R mAb with highest expression level of 2.24 mg/g FW at 5 dpi, with a yield of 21.4 µg/g FW after purification. The purity and N-glycosylation of plant produced antibody was analyzed, including its specificity to human IL-6 receptor by ELISA. Additionally, we investigated the effect to pSTAT3 expression in human PBMC’s by flow cytometry wherein, the results confirmed lower expression of pSTAT3 with increasing concentrations of plant produced anti-hIL-6R mAb. Although, further in vivo studies are key to unveil the absolute functionality of anti-hIL-6R, we hereby show the potential of the plant platform and its suitability for the production of this therapeutic antibody.

## Introduction

The inflammatory cytokine interleukin 6 (IL-6) with its pleiotropic functions, is mostly secreted by monocytes, macrophages, and T cells^1,2^ in response to an array of external stimuli caused by pathogens or tissue injury and other biological substances like cytokines or growth factors^3^. IL-6 interacts with its two receptors namely, membrane-bound IL-6 receptor (mIL-6R) and soluble IL-6 receptor (sIL-6R) to initiate the cellular signaling. mIL-6R is expressed only on macrophages, neutrophils, some types of T-cells and hepatocytes^4^, initiates a classic (cis-) signaling pathway associated with anti-inflammatory response^5^.The sIL-6R present in serum, aids in the initiation of trans-signaling (trans-) thereby stimulating pro-inflammatory responses^6^. After binding of IL-6 with its receptor IL-6R, the complex will in turn bind to gp130 (glycoprotein 130), to further activate JAK/STAT3 (Janus kinase/signal transducer and activator of transcription) pathway and the JAK-SHP2/MAP kinase pathway^7^. Binding of IL-6 to its receptor results in JAK-mediated STAT3 phosphorylation. The dimer of phospho-STAT3 (pSTAT3) translocate to the nucleus for further stimulation of target genes. Rapid and excessive IL-6 production caused by cytokine release syndrome (CRS) promotes tumor invasion, proliferation, and angiogenesis along with the development of an amplifying loop for inflammation^8,9^. Moreover, it was reported that, IL-6 levels rise with disease stage, resulting in respiratory failure and an increased risk of mortality in patients experienced during or after the recent SARS-CoV-2 infection^2,10^.

The suppression of IL-6/JAK/STAT3 signaling has gained emphasis as a therapeutic target for autoimmune diseases and several cancer types associated with immune response disorders characterized by abnormal secretion of inflammatory cytokines. There are several antibodies and drugs that act against IL-6 ligand (E.g. siltuximab, sirukumab, olokizumab, clazakizumab), IL-6R (E.g. tocilizumab, sarilumab, ALX-0061), sgp130 (soluble glycoprotein 130) (E.g. sgp130-Fc, olamkicept), JAKs (E.g. tofacitinib, ruxolitinib, and pacritinib) and STAT3 (e.g. C188-9, OPB-31121, and OPB-51602) which are either approved or in different phases of development and clinical studies^11^. Tocilizumab (TCZ) is a humanized IgG1κ monoclonal antibody against hIL-6R (human interleukin 6 receptor) that was expressed in Chinese Hamster Ovary (CHO) cell line^12^. TCZ has a molecular weight of 149 kDa and is made up of two heavy chains, two light chains^13^ that disrupts IL-6 and IL-6R complex formation by attaching to the IL-6 interaction domain (D2) on IL-6R^14^. This therapeutic antibody was able to inhibit both cis- and trans- IL-6 signaling and has been approved for the treatment of polyarticular and systemic juvenile idiopathic arthritis, giant cell arteritis, Castleman’s disease and rheumatoid arthritis^15^, alongside its efficacy being assessed in the ongoing combination therapeutic trials against cancers^16,17^. Currently, TCZ is also used in the treatment of CRS in COVID-19 affected individuals and found to have a significant recovery of patients with critical illnesses and life posing threats^18^. Evidence from previous reports presented TCZ as an effective candidate in the reduction of pSTAT3 expression without any effect on STAT3 expression in the cytosol^16,19–21^. STAT3 which is an important member of STAT family is mainly activated *via*., phosphorylation by IL-6 receptor signaling^22^ and has a central role in many pathological developments like differentiation, proliferation, survival, inflammation, angiogenesis and immune functions^23–25^ apart from acting as an oncogenic transcription factor^26^. Herein, we pursued to use STAT3 as the biomarker to understand the mechanism of action of plant produced anti-hIL-6R mAb, as it is a direct downstream signal in the IL-6 signaling pathway.

Mammalian cell lines are typically used for mAb expression because of their propensity to carry out proper synthesis, processing and cellular signaling of eukaryotic proteins. They are extremely adaptable and capable of producing glycosylated proteins with post-translational modifications similar to human forms^27–29^. Although this expression system is widely accepted, it has some limitations for instance, there are concerns of contaminations owing to animal viruses, high production cost, low protein yield, requirement of controlled environments^29–32^. Herein, we aim to express a monoclonal antibody using plant platform, that can target the human IL-6 receptor. Advantages of the plant platform include low production costs, high production scale, high production speed and low contamination risk^33,34^. In this study, we produced anti-hIL-6R mAb in *Nicotiana benthamiana* and assessed for its protein assembly, purity and functionality by biochemical methods, immunoassays, and an *in vitro* cell-based assay to monitor pSTAT3 expression.

## Results

### Expression of anti-human interleukin-6 receptor antibody in *N. benthamiana*

**Figure 1:**
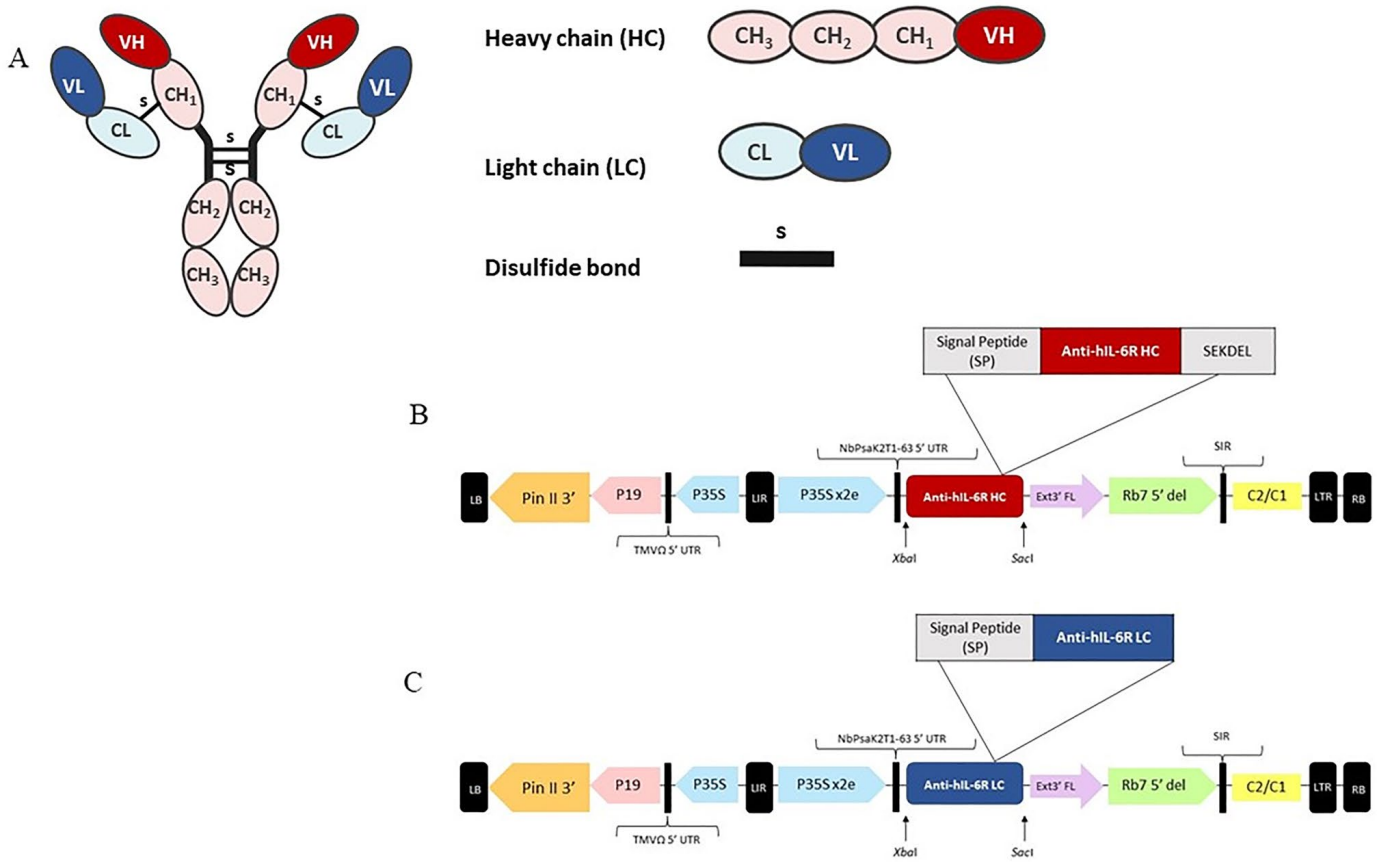
Schematic representation of Monoclonal antibody and geminiviral vector: pBYR2eK2Md (pBYK2e) used in this study. (**A**) Components of monoclonal antibody. The schematic and structural elements assembled in plant produced anti-hIL-6R mAb, T-DNA region of the pBYK2e vector; (**B**) Anti-hIL-6R HC: Heavy chain of anti-hIL-6R antibody; (**C**) Anti-hIL-6R LC: Light chain of anti-hIL-6R antibody.LB and RB: The left and right borders of the T-DNA region transferred by *Agrobacterium* into plant cells; Pin II 3′: The terminator from potato proteinase inhibitor II gene; P19: The RNA silencing suppressor from tomato bushy stunt virus; TMVΩ 5′-UTR: 5′ untranslated region of tobacco mosaic virus Ω; P35S: Cauliflower Mosaic Virus (CaMV) 35S promoter; LIR: Long intergenic region of BeYDV; P35Sx2e: CaMV 35S promoter with duplicated enhancer; NbPsalK2T1-63 5′UTR: 5′ untranslated region; *Xba*I: *Xba*I restriction enzyme site; Signal Peptide (SP): Barley alpha-amylase signal peptide; SEKDEL: Endoplasmic reticulum (ER) retention signal peptide; *Sac*I: *Sac*I-HF restriction enzyme site; Ext3′FL: 3′ region of tobacco (*Nicotiana tabacum*) extension gene; Rb7 5’ del: Tobacco RB7 promoter; SIR: Short intergenic region of BeYDV; C2/C1: Bean Yellow Dwarf Virus (BeYDV) ORFs C1 and C2 encoding for replication initiation protein (Rep) and RepA.

**Figure 2:**
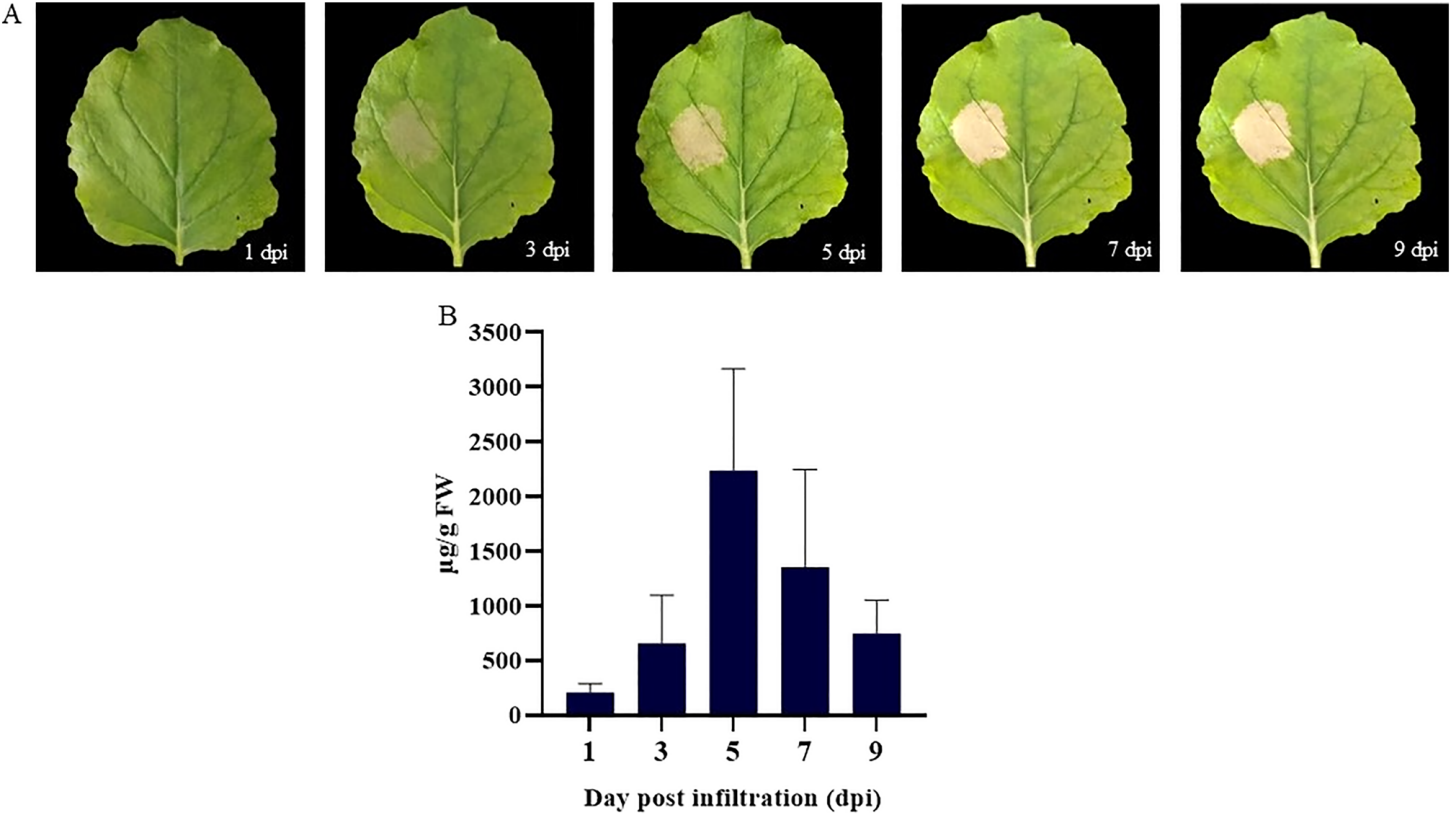
Expression level of plant produced anti-hIL-6R mAb on day-1, 3, 5, 7 and 9 post infiltration in *Nicotiana benthamiana* leaves were quantified by ELISA. The leaf necrosis (**A**) and comparison of anti-hIL-6R mAb yield (**B**) were shown. dpi: days post-infiltration; FW: Fresh Weight; Data are represented as means ± SD of triplicates.

To express anti-hIL-6R antibody in *N. benthamiana*, the V_H_ and V_L_ of anti-hIL-6R mAb were codon-optimized and fused to constant regions of IgG1 heavy chain and light chains respectively. pBY2eK geminiviral plant expression vector was used to clone the light and heavy chains, resulting in pBY2eK-Anti-hIL-6R HC and pBY2eK-Anti-hIL-6R LC (Figure 1). Each construct was transformed into *Agrobacterium tumefaciens* strain GV3101*.* Plant leaves were co-infiltrated with both anti-hIL-6R HC and LC. To confirm the expression of HC and LC, infiltrated leaf samples were analyzed by western blot with anti-human IgG gamma and anti-human kappa detection antibodies (data not shown). Non-infiltrated leaves were used as negative control. For day optimization experiment, leaf samples were harvested on 1-, 3-, 5-, 7- and 9-days post infiltration (dpi). The infiltrated leaves showed the onset of necrosis on 3 dpi with gradual increase until 9 dpi (Figure 2A). ELISA was used for quantifying the expression of plant-produced anti-hIL-6R antibody. The results showed that the maximum expression level of plant-produced anti-hIL-6R was observed at 5 dpi accumulating up to 2.24 mg/g fresh weight (FW) (Figure 2B). The purified protein was accumulated up to 21.4 µg/g of fresh leaf weight.

### Purification of anti-hIL-6R antibody from infiltrated* N. benthamiana* leaves

**Figure 3:**
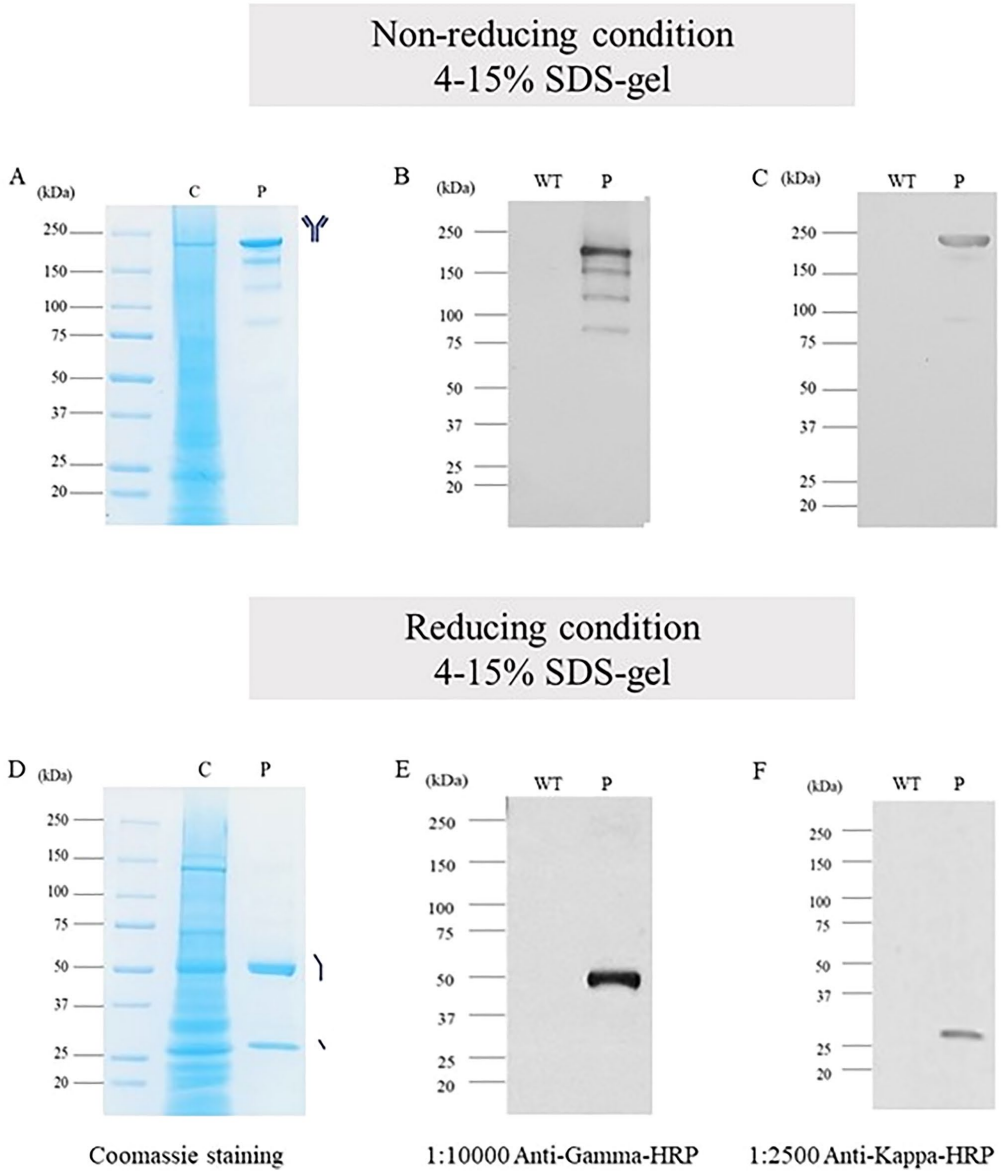
SDS-PAGE and Western blot analysis of plant produced anti-hIL-6R mAb. Non-infiltrated leaves were used as negative control (lane WT). Panels (**A** & **D**), (**B** & **E**) and (**C** & **F**) shows the antibody stained with coomassie, probed with anti-gamma and anti-kappa under non-reducing and reducing condition respectively. C: crude extract 20 µg/lane; P: Purified antibody 5 µg/lane; WT: Wild type.

**Figure 4:**
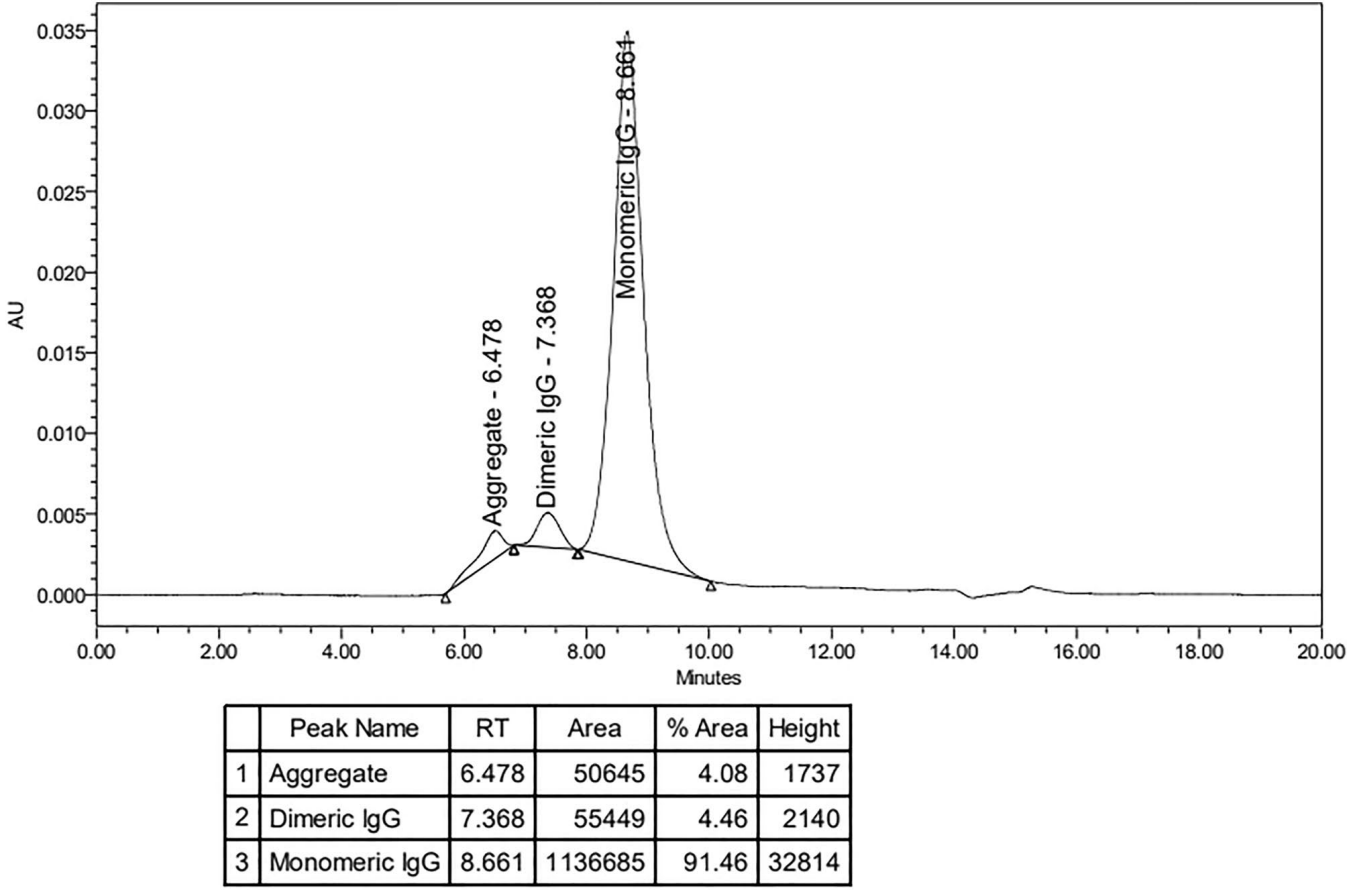
Size exclusion chromatography of plant produced anti-hIL-6R mAb. The chromatogram shows one major and two minor peaks corresponding to monomeric IgG, protein aggregates, dimeric IgG forms respectively.

Agroinfiltrated leaves were harvested, extracted in 1X PBS and centrifuged to obtain the crude extract. The crude extract was purified by Protein-A affinity column chromatography. Non-specific proteins were removed by washing the column with wash buffer and plant produced anti-hIL-6R was eluted by elution buffer. Plant produced anti-hIL-6R antibody was characterized by SDS-PAGE. The expected size under non-reducing condition for anti-hIL-6R HC and LC is approximately 150 kDa (Figure 3A,B,C). Under reducing conditions, the expected molecular weight of anti-hIL-6R-HC is 50 kDa and anti-hIL-6R-LC is 25 kDa. (Figure 3D,E,F) The purified anti-hIL-6R antibody was used for binding assay and in vitro studies. The results of size exclusion chromatography showed that most of the anti-hIL-6R produced by the plant was in the form of full and intact IgG molecules, which accounted for up to 90% of the sample and appeared as the major peak on the chromatogram. However, some smaller amounts of the anti-hIL-6 were found in dimeric (4.46%) and aggregated (4.08%) forms of the sample respectively (Figure 4).

### N-linked glycosylation pattern of anti-hIL-6R antibody

**Figure 5:**
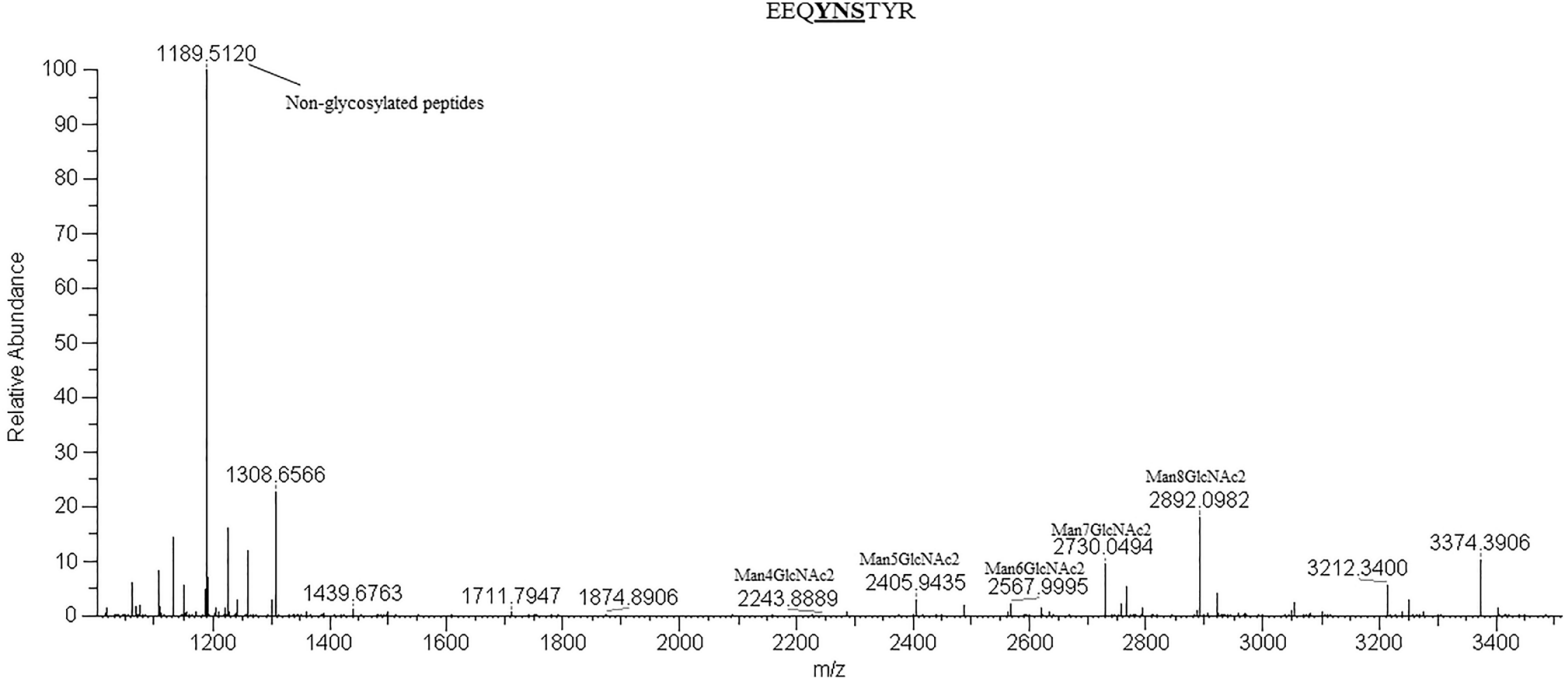
Liquid chromatography-electrospray ionization-mass spectrometry (LC–ESI–MS) of plant produced anti-hIL-6R antibody. The N-glycosylation profiles of the heavy chain peptide EEQYNSTYR (mass: 1189.5120) is shown and the peaks assigned to oligomannosidic N-glycans (Man4-8GlcNAc2) are marked.

To determine the N-glycosylation of anti-hIL-6R antibody produced in wild-type *N. benthamiana*, the purified antibody was subjected to trypsin digestion and analyzed by LC-ESI-MS. The plant produced anti-hIL-6R heavy chain which carries the conserved N-glycosylation site in the Fc region showed the glycosylated peptide EEQYNSTYR with oligomannosidic N-glycans due to the ER retention tag (KDEL) at C-terminal of amino acid sequence (Figure 5). The graphic representation (data shown in supplementary information) of plant produced glycosylated and aglycosylated anti-PD-L1 mAb was used as control.

### Binding activity of plant-produced anti-hIL-6R antibody to human recombinant IL-6R

**Figure 6:**
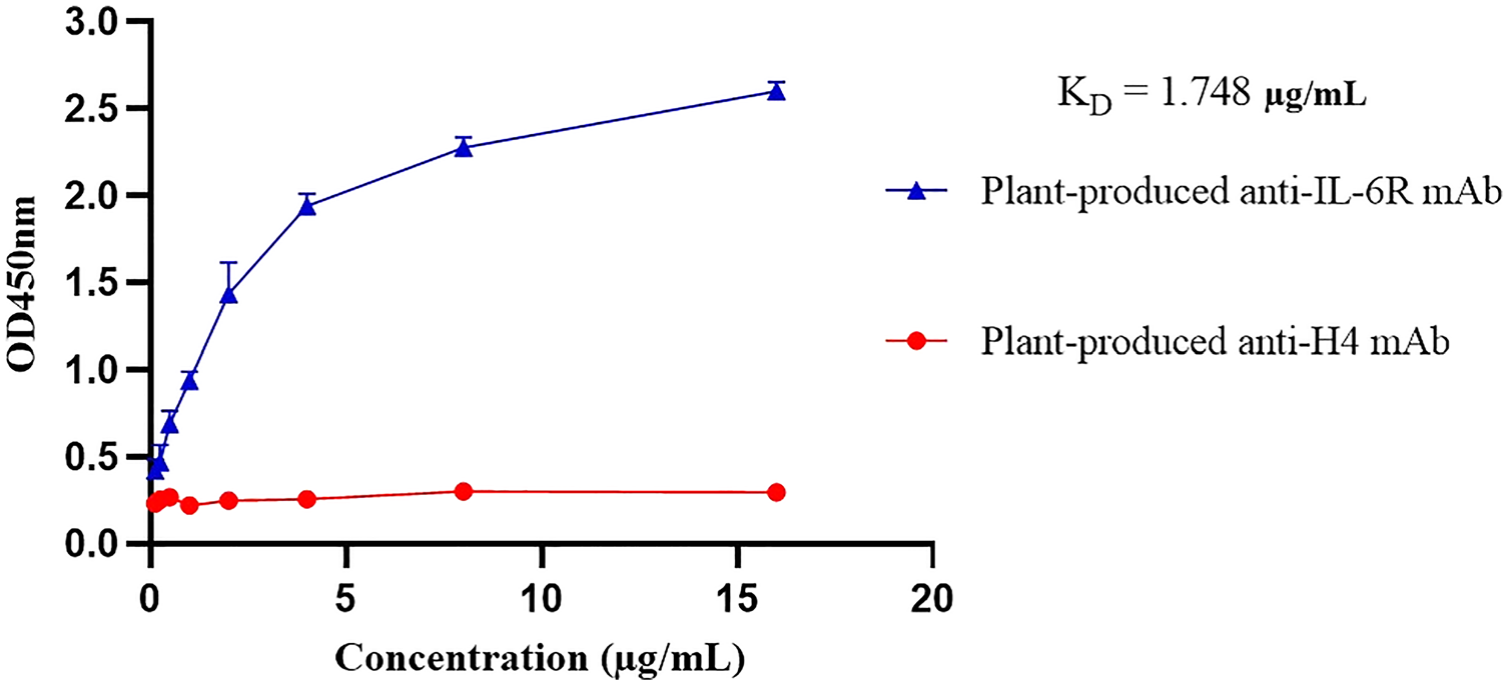
Plant produced anti-hIL-6R binding efficiency to human recombinant interleukin 6 receptor (hIL-6R) by ELISA. Anti-H4 mAb was used as negative control. K_D_: Dissociation constant.

To investigate the specific binding activity of plant-produced anti-hIL-6R, the purified antibody was used in binding ELISA with its target human recombinant IL-6 receptor protein. Plant-produced H4 monoclonal antibody (mAb)^35^ was used as negative control. Each antibody was serially diluted before incubation with hIL-6R. The binding activity was detected by anti-human kappa HRP conjugate (dilution 1:2,500) at OD_450_. The plant-produced anti-hIL-6R antibody elicit specific binding to human recombinant IL-6 receptor protein with a dissociation constant K_D_ value of 1.748 µg/mL (Figure 6), whereas the negative control did not bind as anticipated. Positive control, commercial TCZ was not used in the binding assay due to its limited accessibility in the region^36^. Altogether, the anti-hIL-6R produced in the plant is functional, as indicated by the specific binding to its target.

### In vitro activity of anti-hIL-6R antibody in human PBMCs

**Figure 7:**
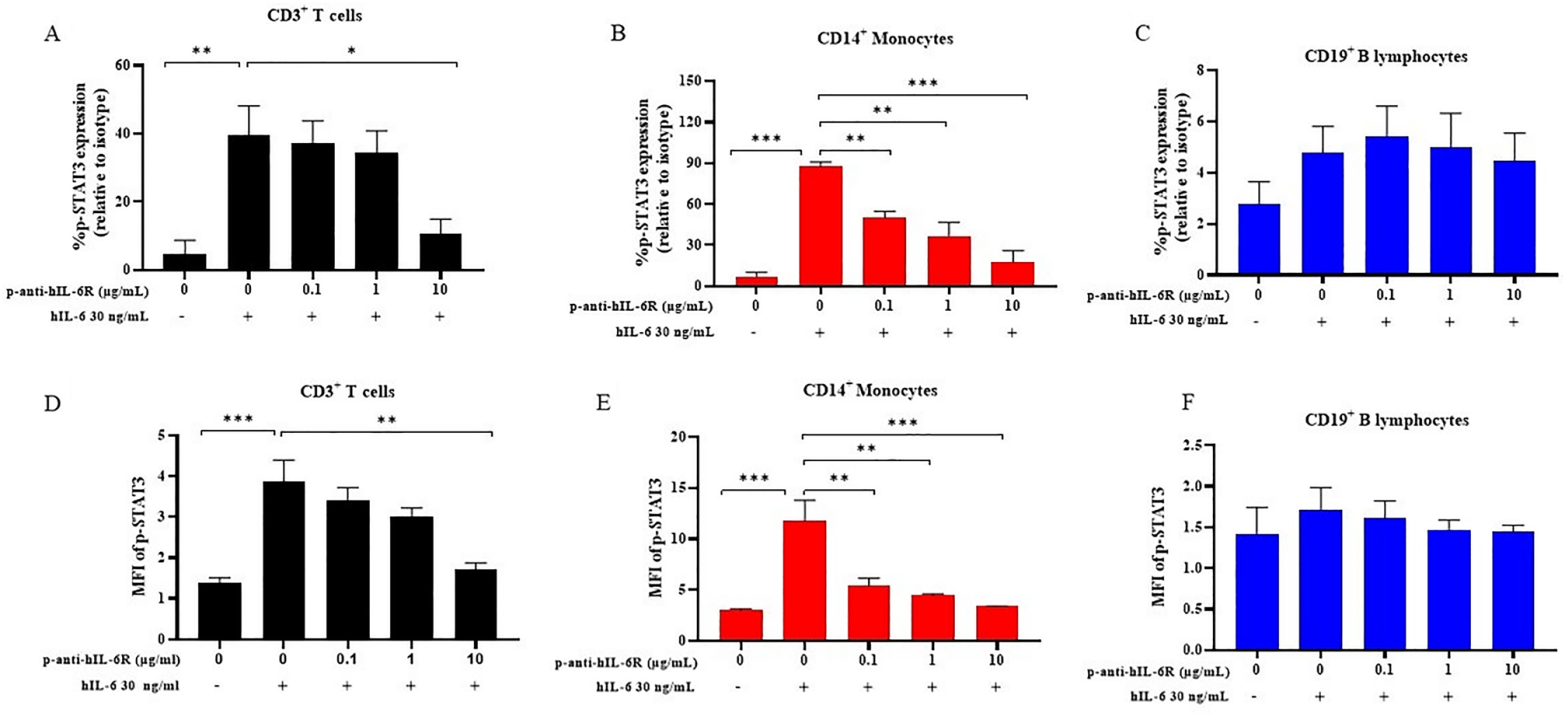
Cell blockade assay of anti-hIL-6R antibody. Whole blood was treated with anti-hIL-6R at a concentration of 0.1, 1, and 10 ug/mL for 20 min, followed by hIL-6 at 30 ng/mL for 15 min. After staining the cells with CD14 (APC-H7) and CD19 (APC), the cells were evaluated using flow cytometry for the pSTAT3 expression. Each value is expressed as the mean ± SEM (n = 3). Dunnett's test, *p < 0.05, **p < 0.01, and ***p < 0.001 versus group with hIL-6 at 30 ng/mL.

PBMCs were isolated from whole blood cells and were investigated for the efficacy of plant produced anti-hIL-6R mAb in suppression of the IL-6/IL-6R/STAT3 pathway by flow cytometry. The pSTAT3 expression and mean flow intensity (MFI) data showed that pSTAT3 expression level was decreased significantly at 10 µg/mL of plant produced anti-hIL-6R mAb in CD3^+^ T cells (Figure 7A,D). Interestingly, 0.1, 1.0 and 10 µg/mL concentrations of plant produced anti-hIL-6R mAb were able to diminish the pSTAT3 expression by almost 2-fold in IL-6 activated CD14^+^ monocytes without much difference (Figure 7B,E). However, there was no significant change in pSTAT3 expression level in CD19^+^ B cells (Figure 7C,F) at all concentrations of plant produced anti-hIL-6R antibody.

## Discussion

Interleukin - 6 is an indication of severe cytokine storm in many cases such as chimeric antigen receptor-T (CAR-T) cell therapy, malignant tumor progression, autoimmune disorders, inflammatory diseases and COVID-19, which could be life-threatening^37,38^. There are many inhibitors that are efficient in the interruption of IL-6 signaling and its pathway^11^. TCZ, a monoclonal antibody targeting human IL-6R, that is approved for treatment of rheumatoid arthritis, Castleman’s disease, juvenile idiopathic arthritis^39^, and newly used for treatment of cytokine release syndrome caused by CAR-T cell therapy^40^ or COVID-19 infection^41^ has gained momentum in the recent few years. Although the drug is effective, the key downside is high treatment expenses due to the involvement of pricey upstream–downstream production technology. The findings by Sinha and Linas, 2021 specifies a total cost of $83,130 in the combination of TCZ and dexamethasone for COVID-19 treatment and were estimated to achieve a gain of 9.36 QALYs (Quality-of-Life-Adjust-Year). Although it was determined that the combination of TCZ and dexamethasone is a cost-effective strategy for persons with COVID-19 who are extremely unwell, it would be important to have a less expensive option that is equally effective as TCZ^42^. Another study in Thailand, reported the arbitration of the price of TCZ currently available on the market to reduce the financial burden of treating refractory systemic juvenile idiopathic arthritis^36^. Hence, the transient expression of recombinant proteins in plants is an alternative, that can overcome the limitations of mammalian cell culture systems which require high manufacturing and scale-up costs, with the risk of contaminations^29,43–46^. Lately, there have been various examples of successful biopharming in plants^47,48^ including virus-like particle (VLP) production as vaccines against influenza H1N1^49^, rotavirus-like particle vaccines^50^, growth factors such as aFGF (acidic fibroblast growth factor)^51^, and antibodies such as mAbs 6D8 (87.2 ± 25.2 μg/g), which is a protective mAb against EBV GP1^52^, anti-CD20-human (2B8) antibody (28 mg/kg); 2B8-Fc-hIL2 immunocytokine (16 mg/kg)^53^, and anti-PD-1 mAb (pembrolizumab)^54^ and diagnostic reagents^55^.

Consequently, we produced monoclonal antibody targeting human IL-6R using transient expression in *N. benthamiana*. Geminiviral vector pBYK2e utilizes the CaMV (cauliflower mosaic virus) 35S promoter for optimal gene expression. The current study showed that anti-hIL-6R mAb was expressed with maximum expression level, accumulating to 2239.92 µg/g fresh weight on 5 dpi (Fig. 2). Previous studies reported that the highest yield of various transiently expressed proteins in *N. benthamiana* at 6 dpi for anti-PD-L1 (atezolizumab) (86.76 µg/g)^56^, anti-IgE (omalizumab) (41.2 µg/g)^57^, B38 (4 µg/g), and H4 mAb (35 µg/g)^35^. Other plant produced proteins expressed showed yields of 18.49 mg/kg of human interleukin-6^58^, 3 mg/kg of cocaine-hydrolase Fc protein^59^, 730 mg/kg of CMG2-Fc protein^60^. These short time periods provide essential advantages of protein expression in terms of speed over other expression approaches including mammalian cells^61–64^ and stable plant expression^65–67^ systems. The significant reduction in antibody expression after 5 dpi might be due to the progressive development of necrosis in the infiltrated leaves. Overexpression of recombinant proteins can result in misfolding of the recombinant proteins, trigger necrosis and subsequent degradation of recombinant proteins^68^.Fine tuning of the purification strategies might mitigate the loss of target protein, thus improving its yield. The N-glycan profile of plant-produced anti-hIL-6R antibody was analyzed by LC–ESI–MS and showed the occurrence of mannosidic oligosaccharides (Man_4–8_GlcNAc_2_) (Fig. 5) which is usually observed at the conserved IgG1-Fc N-glycosylation site of IgG1 immunoglobulin isotype. The KDEL endoplasmic reticulum retention sequence was fused to the heavy chain peptide sequence in anti-hIL-6R mAb expression in *N. benthamiana*^69,70^, to avoid the addition of plant specific glycan sugar moieties during the series of glycosylation reactions in the secretory pathway. Previous studies showed that antibodies with mannosidic N-glycans have increased rates of antibody clearance from circulation^71^, but it is expected that the mannosidic N-glycans have no adverse impact on allergic reactions or hypersensitivity responses^72,73^. The use of glycoengineered plants^70^ and methods to increase the glycosylation efficiency^74^ are alternatives to overcome these bottlenecks for use in potential therapeutic applications.

The specific binding activity of plant-produced anti-hIL-6R mAb to recombinant human IL-6R protein was also investigated by immunoassay. The ELISA results (Fig. 6) of the plant produced anti-hIL-6R mAb was found to have effective binding with human IL-6R, indicating specificity to the target protein whereas plant-produced H4 mAb^35^ showed no binding activity. The K_D_ value reported for mammalian cell-produced tocilizumab is 2.5 nM^75^ and our plant-produced anti-hIL-6R mAb was found to be 1.748 µg/mL. The reason for the difference in binding affinity is currently unclear. The K_D_ value for the mammalian cell-produced antibody was determined using a radioactive cell-based assay employing human myeloma cell line^76^, while this study employed ELISA to assess the affinity of plant produced anti-hIL-6R to the target protein. Due to the limited access for commercial TCZ (control), we could not directly compare the mammalian TCZ with the plant produced variant in our binding and in vitro assays. Occurrence of protein aggregates as evident from size exclusion chromatography, might also have interference in the binding activity.  However, the study by Heinrich et al., 2010 shows that kinetic constants characterized for antibody interaction to its respective receptors by different methods are discrete, as the principle varies in each biophysical technique adopted.^77^.

To determine the biological function of plant-produced anti-hIL-6 mAb, we performed the inhibitory effect of the mAb effect on STAT3 phosphorylation. Our results demonstrated that pSTAT3 expression in CD14^+^ cells (monocytes and macrophages) and CD3^+^ T cells was decreased, whereas almost no change was found in CD19^+^ (B cells). A study by Zhang et al., 2020b showed that the decrease in pSTAT3 expression in CD3^+^ T cells and CD14^+^ monocytes from human PBMCs depends on the concentration of anti-hIL-6R mAb or tocilizumab, which is consistent with our findings using plant produced anti-hIL-6R mAb in these two cell populations. The data showed that tocilizumab completely repressed the pSTAT3 expression in CD3^+^ T cells and CD14^+^ monocytes at 12.5 µg/mL^78^ which is similar to our plant produced anti-hIL-6R mAb at 10 µg/mL. Activation of IL-6R/JAK/STAT3 signaling has been shown to induce systemic hyperinflammation during cytokine storm^79^. In addition, IL-6/JAK/STAT3 signaling has been demonstrated to play important roles in tumor progression and aggressiveness in several cancers^11,26,80–84^. Hence, STAT3/pSTAT3 was chosen as the biomarker in this preliminary study. In addition, our study results are analogous with sarilumab, an anti-IL-6R mAb expressed in *N. benthamiana* in certain characteristics that was evident from the study by Jugler et al., 2021^85^. However, further research including in vivo studies in animal models must be conducted to compare the efficacy of our plant produced mAb with commercial anti-hIL-6R mAb.

## Conclusion

IL-6 is a hallmark cytokine of CRS that can impose life-threatening response events. Although there are various IL-6 signaling inhibitors approved in the market, the production and treatment costs are high-priced. Altogether, we produced a recombinant anti-hIL-6R mAb that can recognize human IL-6 receptor using plant as an expression platform. The results of plant produced anti-hIL-6R mAb demonstrate specific binding to human IL-6 receptor, in vitro inhibition of IL-6:IL-6R complex formation and decreased STAT3 expression. Further, to reveal the effectiveness of anti-hIL-6R mAb produced in plants, animal studies need to be performed for better insights on its functioning in the IL-6 signaling pathway and disease progressions.

## Methods

### Study details

The study was permitted to be carried out by the internal CU-IBC (Chulalongkorn University-Institutional Biosafety Committee) following all the Biosafety guidelines for Modern biotechnology. All methods were performed in accordance with the relevant guidelines/regulations/legislation. The seeds of *N. benthamiana* used in the present study were kindly gifted by Dr. Supaart Sirikantaramas, Faculty of Science, Chulalongkorn University.

### Construction of plant expression vector

The variable region of heavy chain (V_H_) and light chain (V_L_) from previous study report^86^ were codon-optimized to *N. benthamiana* using Invitrogen GeneArt® Gene Synthesis (Thermo Fisher Scientific) and commercially synthesized (Genewiz, Suzhou, China). The synthesized V_H_ and V_L_ were fused to human IgG1 constant regions of heavy chain (C_H_ and light chain (C_L_) and were flanked with signal peptide on the N-terminus respectively and a SEKDEL (Ser-Glu-Lys-Asp-Glu-Leu) sequence on C-terminus of heavy chain. Further, they were ligated to pBYR2eK2Md (pBYK2e) geminiviral expression vector to make pBYK2e-Anti-hIL-6R heavy chain (HC) and pBYK2e-Anti-hIL-6R light chain (LC). Each ligation product was transformed into *Escherichia coli* DH10B strain via., heat shock and confirmed by colony PCR (polymerase chain reaction). Subsequently, the extracted plasmid was transformed into *Agrobacterium tumefaciens* (GV3101) via., electroporation.

### Transient expression and day optimization of Anti-hIL-6R antibody in *N. benthamiana*

*N. benthamiana* plants are grown approximately for 6–8 weeks before using for agroinfiltration. Agrobacterium cells containing either pBYK2e-Anti-hIL-6R HC or pBYK2e-Anti-hIL-6R LC were cultured with continuous shaking at 200 rpm at 28 °C for 20 h. Overnight, Agrobacterium cultures (HC, LC) were centrifuged at 5,000 × g for 8 min, and the bacterial pellets were resuspended in 1X infiltration buffer to get a final OD_600_ of 0.2. The same amount of HC and LC harboring Agrobacterial cultures were mixed in the ratio 1:1 and syringe infiltrated into *N. benthamiana* leaves. The infiltrated plants were incubated in a controlled environment. To determine small scale protein expression level, the leaf discs from infiltrated leaves were collected on 1-, 3-, 4-, 5-, 6-, and 7 days post infiltration (dpi) in 1.5 mL centrifuge tubes.

### Protein extraction and purification

Infiltrated leaves were harvested and extracted in 1X PBS (phosphate-buffered saline: 137 mM NaCl, 2.7 mM KCl, 4.3 mM Na_2_HPO_4_, 1.47 mM KH_2_PO_4_, pH 7.4) in the presence of stainless-steel beads using a tissue homogenizer. The leaf extracts were centrifuged at 13,000 rpm for 15 min and the supernatant/crude extract collected was used for protein quantification and expression analysis. After the maximum expression of anti-hIL-6R antibody was assessed in the day optimization experiment, large scale vacuum infiltration was performed and harvested leaves were used for extraction in a blender with 1X PBS. The similar steps were followed as mentioned above before the large-scale purification of the anti-hIL-6R protein.

For purification, the plant leaves infiltrated on large-scale using vacuum infiltration were harvested on the day before expression yield analysis. The harvested leaves were blended in 1X PBS extraction buffer in the ratio of 1 g of leaf:3 mL of buffer. Then, the crude extract was filtered using a gauze cloth, centrifuged at 15,000 × g for 30 min at 4 °C and the supernatant was again filtered through 0.22 µm membrane filter. The protein-A affinity chromatography was employed for protein purification. Crude extract was loaded onto the Protein-A resin column. The column was washed with 5X column volumes of 1X PBS. 0.1 M glycine buffer at pH 2.7 was used to elute the target anti-hIL-6R antibody and further neutralized with 1.5 M Tris–HCl at pH 8.8.

### Analysis of anti-hIL-6R antibody by SDS-PAGE

The expression and purity of anti-hIL-6R antibody was analyzed using SDS-PAGE (sodium dodecyl sulphate polyacrylamide gel electrophoresis) and western blotting. In brief, approximately 20 µg of protein samples were mixed separately in 10X non-reducing (125 mM Tris–HCl pH 6.8, 12% Sodium Dodecyl Sulphate 10% Glycerol, 0.001% Bromophenol blue) and reducing (125 mM Tris–HCl pH 6.8, 12% Sodium Dodecyl Sulphate 10% Glycerol, 22% β-mercaptoethanol, 0.001% Bromophenol blue) dyes, heated at 95 °C for 5 min. Samples were then loaded on 4–15% gradient SDS polyacrylamide gel along with pre-stained SDS-PAGE standard (Bio-Rad, California, USA). Electrophoresis was performed at room temperature for 80 min using a constant voltage of 120 V in 1X running buffer (25 mM Tris, 192 mM Glycine, 1% SDS) until the dye front reached the end of the gel.

### Protein staining and western blot

SDS-PAGE gels were stained using Instantblue Coomassie stain (Abcam, UK). Gels were washed three times in deionized water for 5 min, incubated in 10 mL of Coomassie blue stain for 4 h, de-stained 2–3 times in 10 mL of deionized water for 1 h, until clear protein bands were visualized. For western blotting, the electrophoretic separated proteins from the gel were subsequently transferred to a nitrocellulose membrane (Bio-Rad, California, USA) using 1X transfer buffer (25 mM Tris, 192 mM Glycine, 15% Methanol) at 100 V for 2 h. The nitrocellulose membrane after washing with deionized water, was blocked with 5% skim milk in 1X PBS for 30 min followed by 1X PBST (0.05% Tween 20 in 1X PBS) washes. The membrane was incubated either with 1:10,000 conjugated anti-human IgG gamma or with 1:2,500 conjugated anti-human IgG kappa antibody diluted in 1X PBS and 3% skim milk. After further washes, enhanced chemiluminescence (ECL) plus detection reagent (Abcam, UK) was used to record and capture the plant-expressed protein signals on nitrocellulose membrane to a light-sensitive X-ray film (Carestream, New York, USA).

### Size exclusion chromatography of anti-hIL-6R antibody

5 µg of monoclonal antibody was injected into a UHPLC system (Waters, MA, USA) equipped with a BEH SEC 200 column (4.6 mm × 300 mm, Waters, MA, USA). The protein was eluted from the column using 1X PBS buffer (pH 7.4) at a flow rate of 0.3 mL/min, while the column was maintained at 25 °C, and the UV detector was set at 280 nm. The peak areas were automatically integrated using Empower3 software (Waters, MA, USA).

### N-glycan analysis

The N-linked glycosylation of anti-hIL-6 antibody was analyzed by liquid chromatography-electrospray ionization-mass spectrometry (LC–ESI–MS). The purified anti-hIL-6 antibody was run on SDS-PAGE under reducing conditions and then stained with Instant Blue Coomassie protein–dye (Abcam, UK) to view the separated antibody chains. Plant-produced anti-hIL-6 heavy chain was excised from the gel, S-alkylated and then digested with trypsin. The tryptic peptides were analyzed on a Vanquish™ Neo UHPLC (ThermoFisher Scientific, USA) system coupled to an Orbitrap Exploris 480 mass spectrometer (ThermoFisher Scientific, USA). The positive glycopeptides of m/z = 350–3200 were monitored and captured on the chromatogram. The expected glycopeptides on EEQYNSTYR site were manually detected and analyzed with FreeStyle 1.8 program (Thermo Scientific, USA).

### Anti-hIL-6R antibody quantification by ELISA

The expression yield of crude and purified plant-produced anti-hIL-6R was determined by enzyme-linked immunosorbent assay (ELISA). A 96-half well microplate (Costa® Assay Plate, Corning, USA) was coated with 25 µL/well of anti-human IgG Fc fragment (Abcam, Cambridge, UK) diluted at a ratio of 1:1,000 in 1X PBS and incubated at 4 °C overnight. After incubation, the plate was washed with 170 µL/well of 1X PBS-T (1X PBS with 0.05% Tween-20) for 3 times and blocked with 5%(w/v) skim milk in 1X PBS at 37 °C for 2 h. The commercially available human IgG1 isotype control (Abcam®, Cambridge, UK), diluted twofold in 1X PBS starting at a concentration of 1 µg/mL was used as standard. Plant crude extracts and purified samples were used at a dilution of 1:1,000 and 1:2,000; respectively in 1X PBS. After washing, each dilution of the standard and samples were added into the microplate in duplicates. Then the microplate was incubated at 37 °C for 2 h followed by 3X washes with 1X PBS-T. The plate was then incubated with peroxidase-conjugated goat anti-human kappa antibody (Southern Biotech, Birmingham, USA) at 1:2,500 dilution in 1X PBS. After washing, the plate was developed by adding 25 µL/well of TMB (3,3′,5,5′-tetramethylbenzidine) (Promega®, USA) and incubated for 5 min. The reaction was stopped with 25 µL/well of 1 M H_2_SO_4_. Ultimately, the absorbance was measured at 450 nm in a 96-well plate reader (BMG Labtech, Germany). The standard curve was generated, and the concentration of samples were calculated from linear equation (y = mx + c). The experiments were performed in triplicates and data are presented as mean ± standard deviation (SD).

### Specificity of anti-hIL-6R

Plant-produced anti-hIL-6R antibody binding activity was investigated by ELISA using specific human IL-6 receptor (hIL-6R) recombinant protein. Three biological replicates were performed. Each 96 half well in the microplate was coated with 25 µL of 2 µg/mL human IL-6R (Sino Biological, USA) and incubated at 4 °C overnight. The incubated plate was washed with 1X PBS-T (3 times) and blocked with 5%(w/v) non-fat milk in 1X PBS at 37 °C for 2 h. The plate was further incubated with plant-produced anti-hIL-6R samples at 37 °C for 2 h. After washing, the plate was treated with goat anti-human kappa-HRP (1:2,500) (Southern Biotech, Birmingham, USA) for 1 h at 37 °C. Finally, the plate was washed and incubated with 25 µL/well of 3,3′,5,5′-tetramethylbenzidine (TMB) (Promega®, USA). The reactions were stopped by addition of 25 µL/well of 1 M H_2_SO_4_ and the absorbance was read at 450 nm by microplate reader (Hercuvan system, UK). Plant-produced H4 mAb^35^ was used as negative control. The data were analyzed by GraphPad Prism 9.3 software (San Diego, CA, USA). The dissociation constant (K_D_) was determined by non-linear regression analysis using a one-site binding model.

### Effect of plant produced anti-hIL-6R mAb in vitro

Healthy individuals (n = 3) were recruited following written informed consent. The study was approved by the institutional review boards of the participating centers (Faculty of Medicine, Chulalongkorn University, COA No.1371/2022, IRB. Approval No. 633/64) and the experimental methods comply with the Helsinki Declaration. Briefly, healthy whole blood cells were incubated with plant produced anti-IL-6R antibody and was stimulated with human IL-6 (Sino Biological, USA). To lyse the cells, 1X Lysis buffer (BD Pharm Lyse Solution, BD Bioscience, USA) was added and incubated and the reaction was stopped by addition of 1X PBS. Then, the samples were incubated with APC Mouse Anti-Human CD19 (BD Pharmingen USA), and APC-H7 Mouse Anti-Human CD14 (BD Pharmingen USA). The samples were centrifuged, and the pellet was resuspended in fixation buffer (BD Pharmingen, USA) and followed by 2 washes with of staining buffer. Each sample was incubated with Perm Buffer III (BD Phosflow, BD Bioscience, USA), washed twice with staining buffer. Anti-STAT3 Phospho (Tyr705) antibody (PE, Biolegend, USA) or Mouse IgG1κ isotype antibody (PE, Biolegend, USA) was added and incubated in dark at room temperature for 30 min. Each sample was washed with staining buffer twice. Flow cytometry was performed to analyze the results. Post hoc Dunnett’s test was used for statistical analysis in GraphPad Prism 9.3 software (San Diego, CA, USA).

### Statistical analysis

All the values were expressed as mean ± SD for duplicate samples in two biological replicates. Post hoc Dunnett’s test was used for statistical analysis in flow cytometry.

## Supplementary information

Raw gel pictures and Glycan profiles of control antibody are provided in the supplementary file.

### Supplementary Information

Supplementary Information.

## Data Availability

The original contributions presented in the study are included in the article, further inquiries can be directed to the corresponding author.
