# Author Correction: Effect of plant produced Anti-hIL-6 receptor antibody blockade on pSTAT3 expression in human peripheral blood mononuclear cells

**DOI:** 10.1038/s41598-023-41344-6

**Published:** 2023-08-29

**Authors:** Namthip Kaewbandit, Ashwini Malla, Wanuttha Boonyayothin, Kaewta Rattanapisit, Thareeya Phetphoung, Nuttapat Pisuttinusart, Richard Strasser, Rattana Saetung, Supannikar Tawinwung, Waranyoo Phoolcharoen

**Affiliations:** 1 Center of Excellence in Plant-Produced Pharmaceuticals, Chulalongkorn University, Bangkok, Thailand; 2 Department of Pharmacognosy and Pharmaceutical Botany, Faculty of Pharmaceutical Sciences, Chulalongkorn University, Bangkok, Thailand; 3 Graduate Program of Pharmaceutical Sciences and Technology, Faculty of Pharmaceutical Sciences, Chulalongkorn University, Bangkok, Thailand; 4 Baiya Phytopharm Co., Ltd, Bangkok, Thailand; 5 Department of Applied Genetics and Cell Biology, University of Natural Resources and Life Sciences, Vienna, Austria; 6 Department of Pharmacology and Physiology, Faculty of Pharmaceutical Sciences, Chulalongkorn University, Bangkok, 10330 Thailand; 7 Cellular Immunotherapy Research Unit, Chulalongkorn University, Bangkok, Thailand

Correction to: *Scientific Reports* 10.1038/s41598-023-39106-5, published online 24 July 2023

The Acknowledgements section in the original version of this Article was incomplete. It now reads:

This Research is funded by the Thailand Science Research and Innovation Fund Chulalongkorn University (BCG66330001). The author N.K. is a recipient of the graduate school master’s degree program scholarship, to commemorate the 72nd anniversary of His Majesty King Bhumibol Adulyadej. The MS equipment was kindly provided by the BOKU Core Facility Mass Spectrometry (MS), and we thank Rudolf Figl for technical assistance with the MS analysis.

The original Article has been corrected.

