# Supplementary material for: Effect of plant produced Anti-hIL-6 receptor antibody blockade on pSTAT3 expression in human peripheral blood mononuclear cells
Source: Sci Rep. 2023 Jul 24;13:11927. doi: 10.1038/s41598-023-39106-5 (PMC10366097; doi:10.1038/s41598-023-39106-5)

Effect of Plant Produced Anti-hIL-6 Receptor Antibody Blockade on pSTAT3 Expression in Human Peripheral Blood Mononuclear Cells

**Namthip Kaewbandit^1,2,3^, Ashwini Malla^4^, Wanuttha Boonyayothin^2,3^, Kaewta Rattanapisit^4^, Thareeya Phetphoung^2,3^, Nuttapat Pisuttinusart^2,3^, Richard Strasser^5^,** **Rattana Saetung^6^, Supannikar Tawinwung^*6,7^, Waranyoo Phoolcharoen^1,2*^**

^1^Center of Excellence in Plant-produced Pharmaceuticals, Chulalongkorn University, Bangkok, Thailand.

^2^Department of Pharmacognosy and Pharmaceutical Botany, Faculty of Pharmaceutical Sciences, Chulalongkorn University, Bangkok, Thailand.

^3^Graduate Program of Pharmaceutical Sciences and Technology, Faculty of Pharmaceutical Sciences, Chulalongkorn University, Bangkok, Thailand.

^4^Baiya Phytopharm Co., Ltd., Bangkok, Thailand.

^5^Department of Applied Genetics and Cell Biology, University of Natural Resources and Life Sciences, Vienna, Austria.

^6^Department of Pharmacognosy and Pharmaceutical Botany, Faculty of Pharmaceutical Sciences, Chulalongkorn University, Bangkok 10330, Thailand.

^7^Cellular Immunotherapy Research Unit, Chulalongkorn University, Bangkok, Thailand.

[*Waranyoo.P@chula.ac.th](mailto:*Waranyoo.P@chula.ac.th)

[*Supannikar.t@pharm.chula.ac.th](mailto:*Supannikar.t@pharm.chula.ac.th)


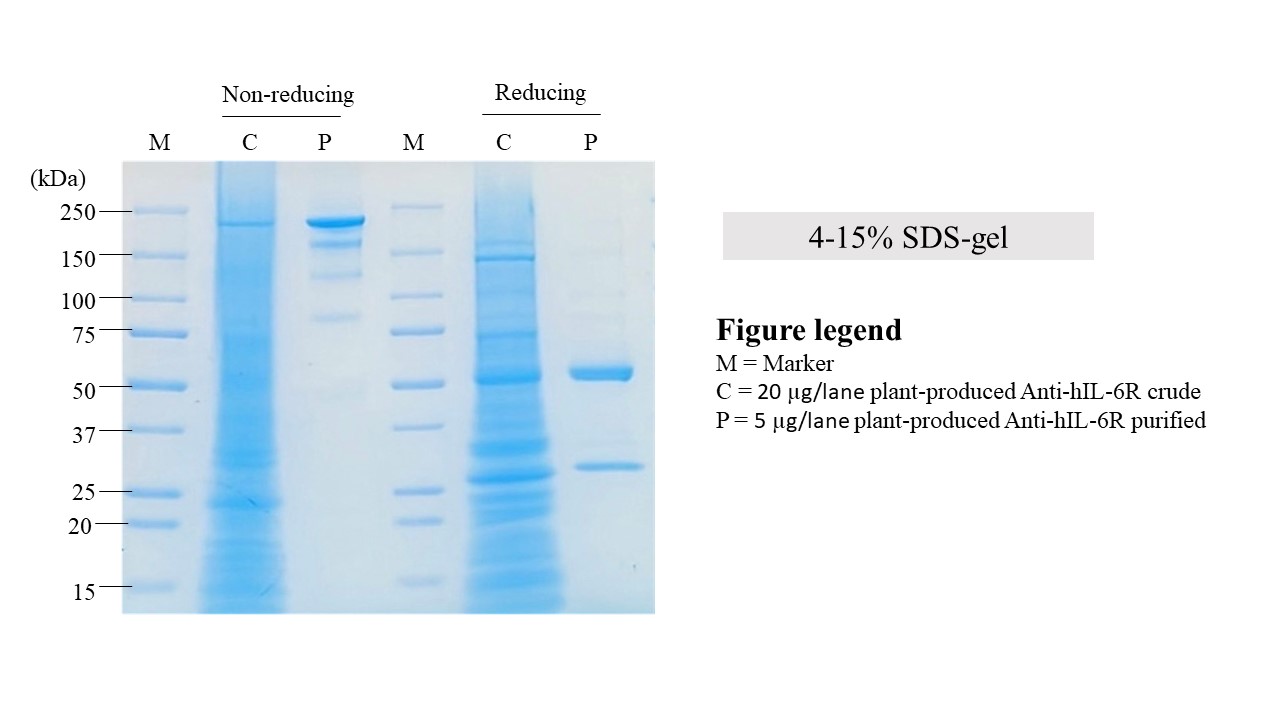


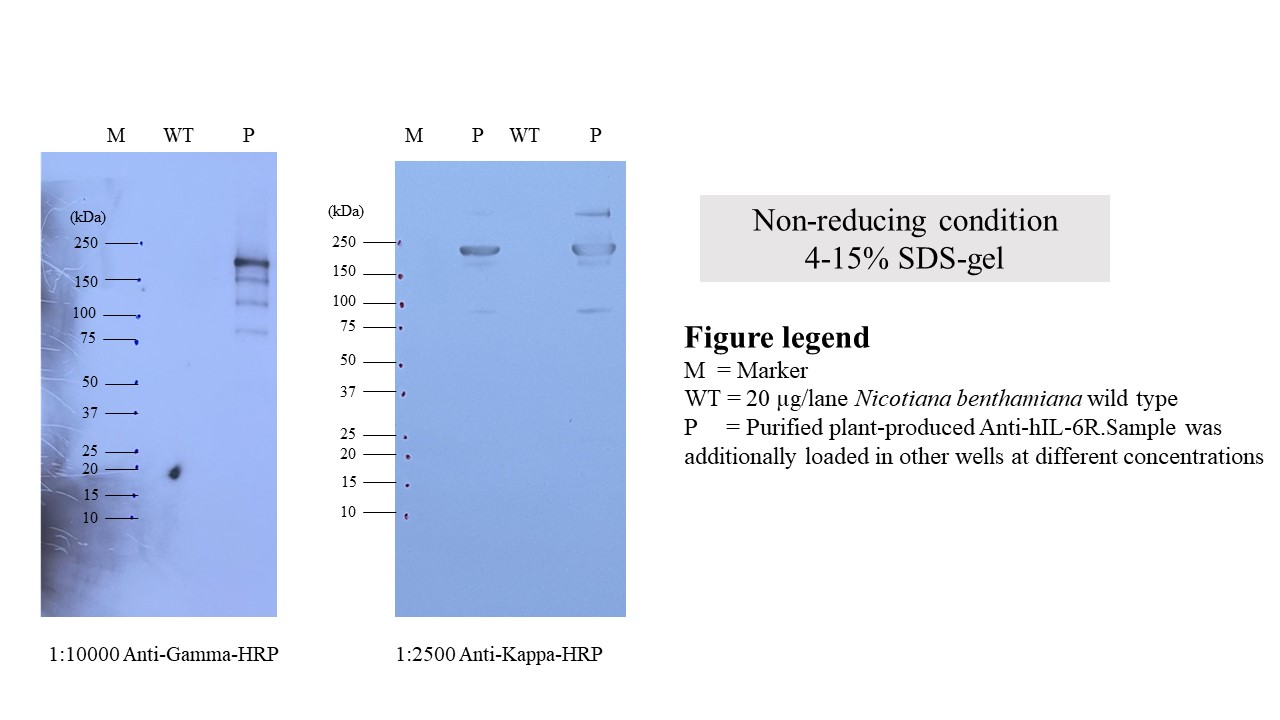


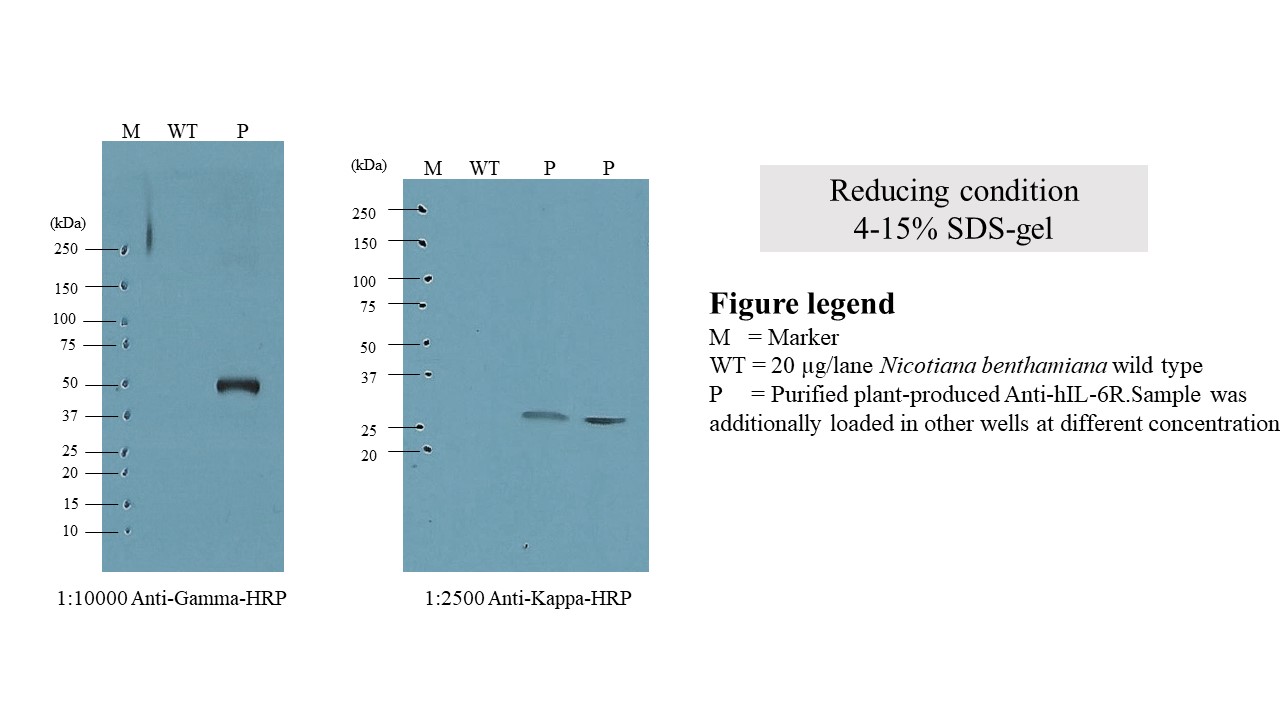


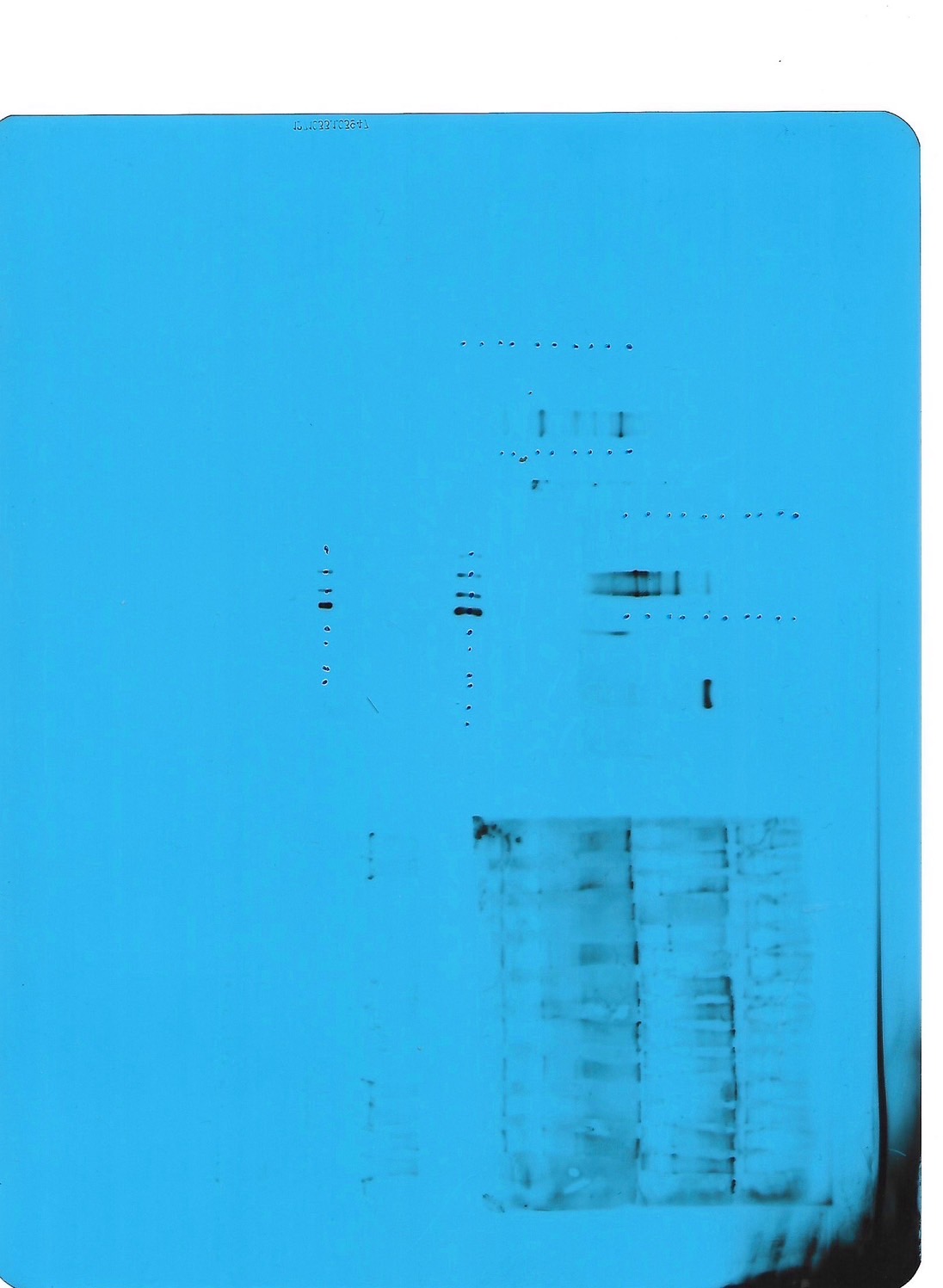


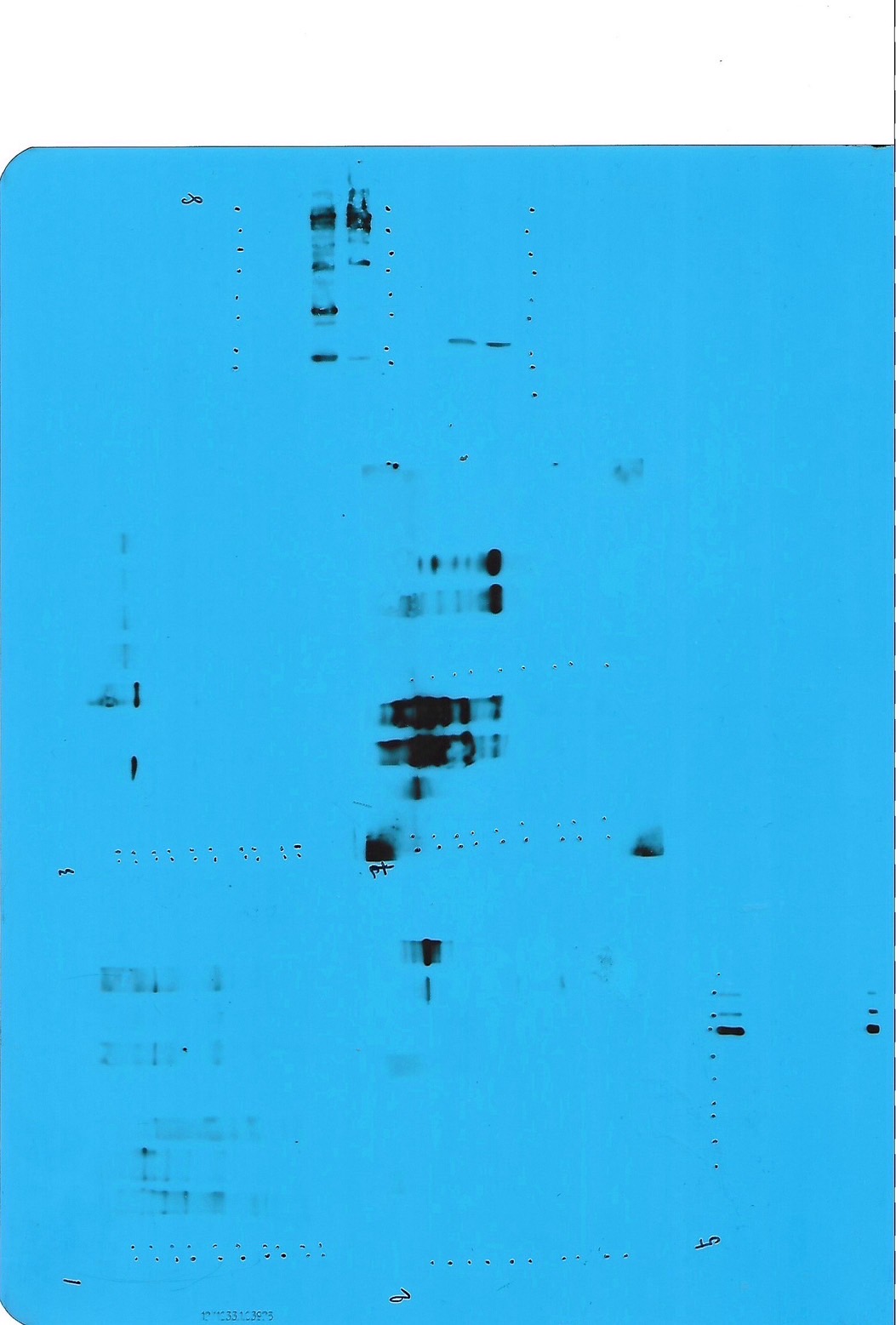


**Glyco-profiles of Plant Produced Anti-PD-L1 and Engineered Anti-PD-L1**


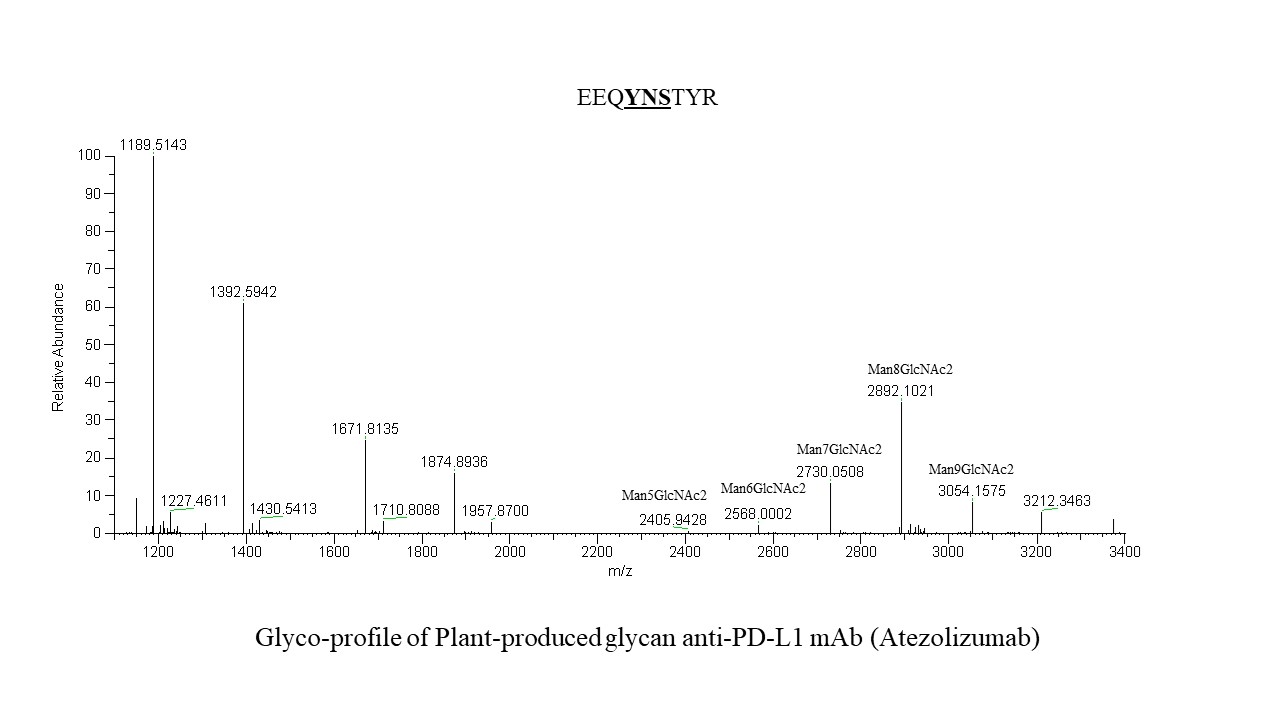

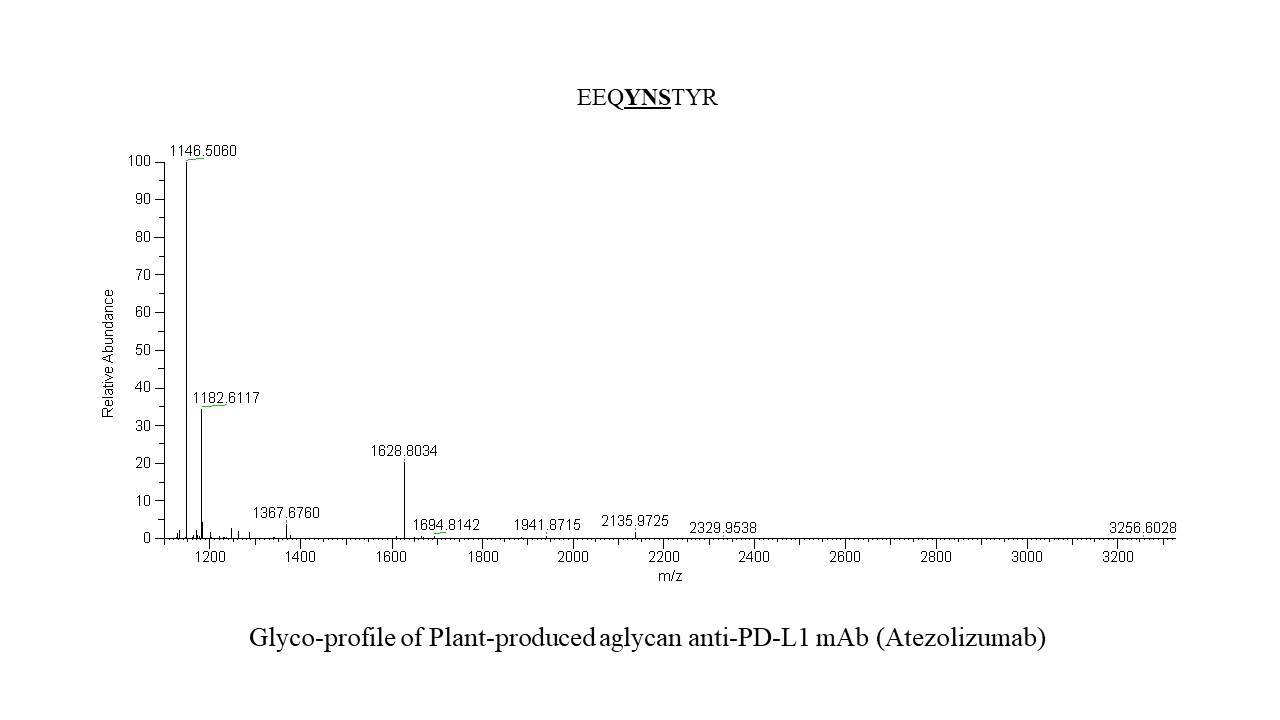

Supplement: Supplementary file 1 — Supplementary Information. [file 41598_2023_39106_MOESM1_ESM.docx]
